# Supplementary material for: Association of Plasma Placental Growth Factor with White Matter Hyperintensities in Alzheimer’s Disease
Source: Biomolecules. 2025 Sep 26;15(10):1367. doi: 10.3390/biom15101367 (PMC12564238; doi:10.3390/biom15101367)
Supplement: Supplementary file 1 [file biomolecules-15-01367-s001.zip › PlGF_Supplementary Figure_20250922.pptx]

## Slide 1
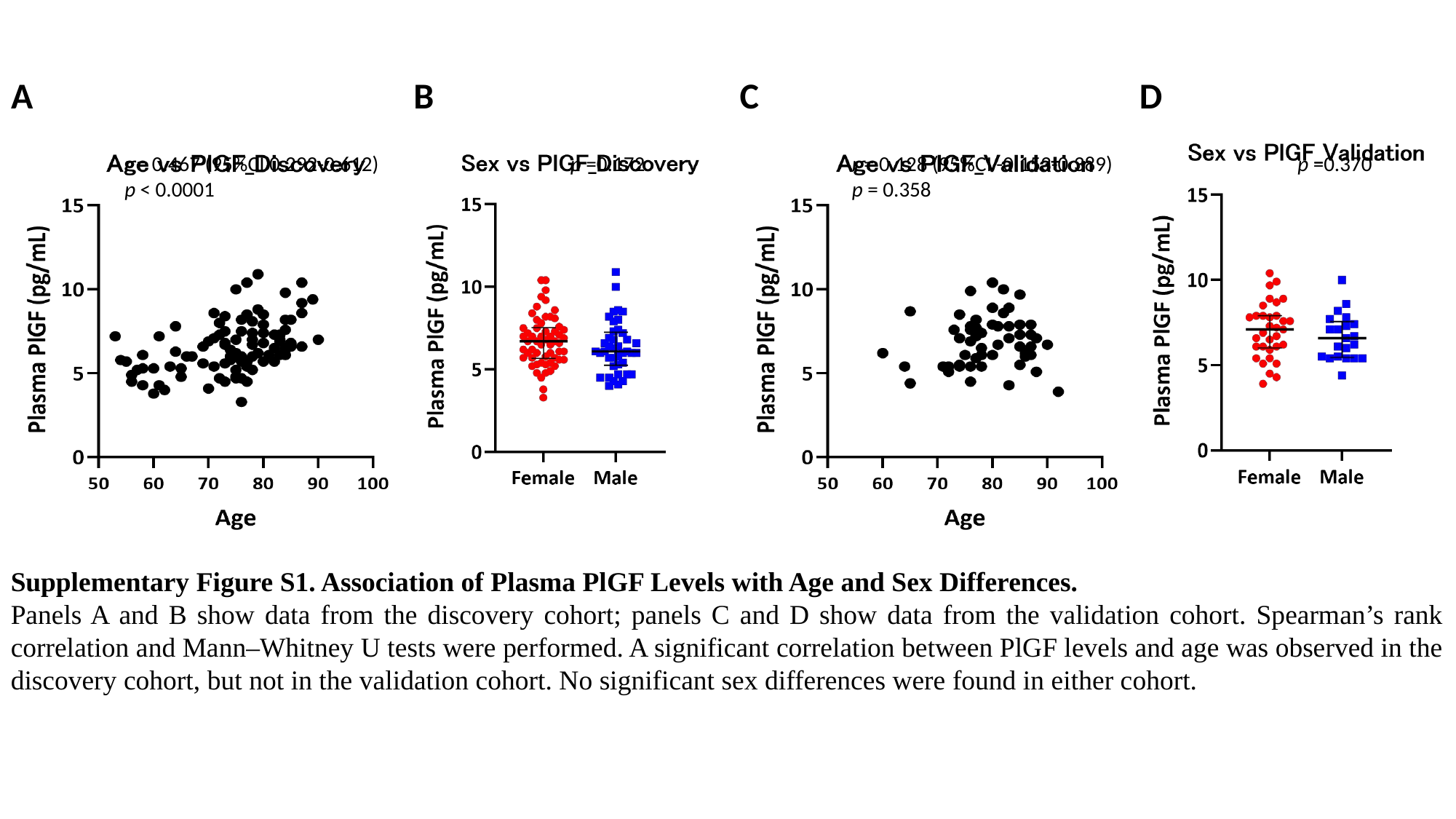

A
B
C
D
r = 0.467 (95%CI 0.292-0.612)
p < 0.0001
p =0.172
r = 0.128 (95%CI -0.153-0.389)
p = 0.358
p =0.370
Supplementary Figure S1. Association of Plasma PlGF Levels with Age and Sex Differences.
Panels A and B show data from the discovery cohort; panels C and D show data from the validation cohort. Spearman’s rank correlation and Mann–Whitney U tests were performed. A significant correlation between PlGF levels and age was observed in the discovery cohort, but not in the validation cohort. No significant sex differences were found in either cohort.

## Slide 2
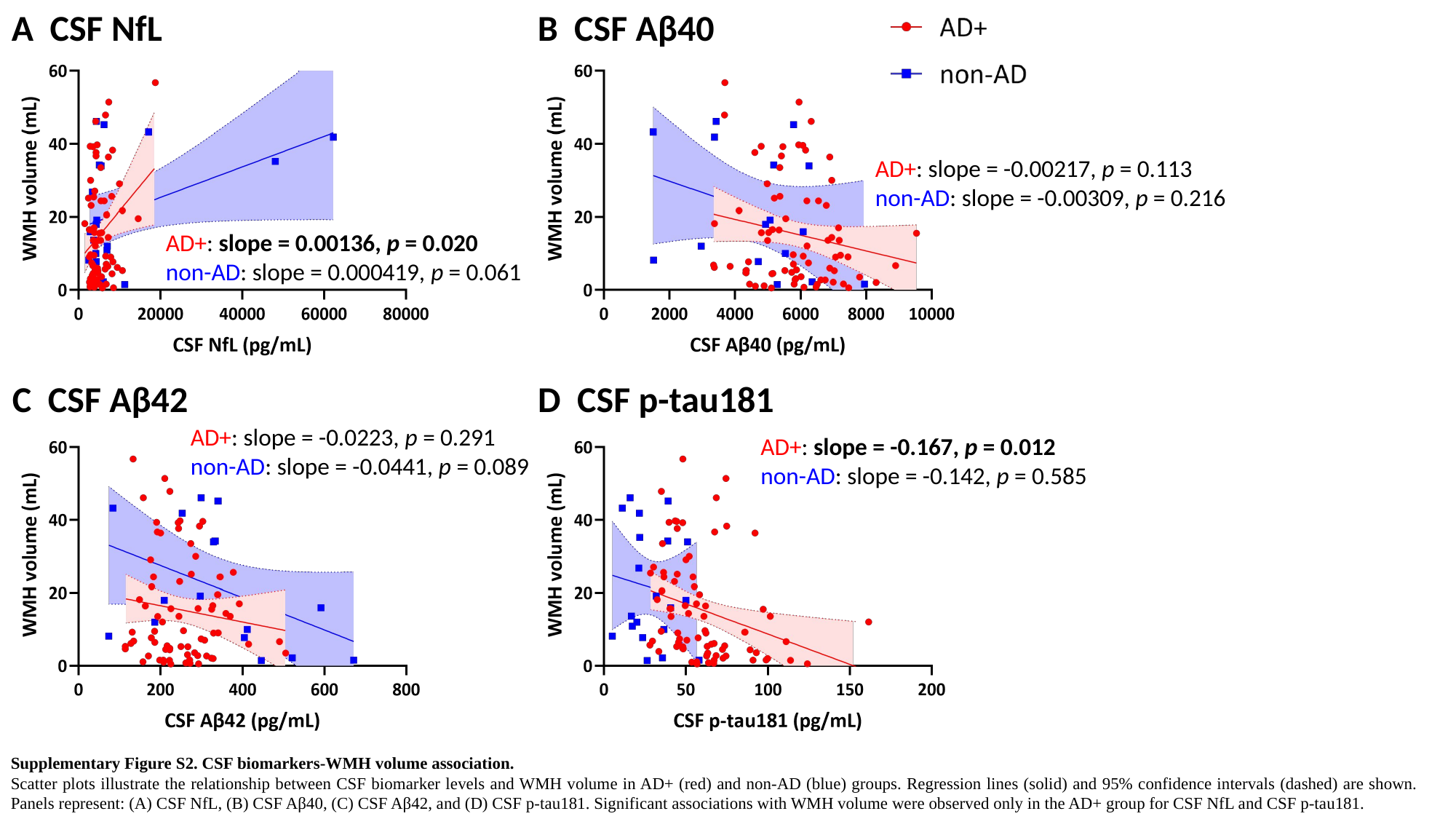

A CSF NfL
B CSF Aβ40
AD+: slope = -0.00217, p = 0.113
non-AD: slope = -0.00309, p = 0.216
AD+: slope = 0.00136, p = 0.020
non-AD: slope = 0.000419, p = 0.061
C CSF Aβ42
D CSF p-tau181
AD+: slope = -0.0223, p = 0.291
non-AD: slope = -0.0441, p = 0.089
AD+: slope = -0.167, p = 0.012
non-AD: slope = -0.142, p = 0.585
Supplementary Figure S2. CSF biomarkers-WMH volume association.
Scatter plots illustrate the relationship between CSF biomarker levels and WMH volume in AD+ (red) and non-AD (blue) groups. Regression lines (solid) and 95% confidence intervals (dashed) are shown. Panels represent: (A) CSF NfL, (B) CSF Aβ40, (C) CSF Aβ42, and (D) CSF p-tau181. Significant associations with WMH volume were observed only in the AD+ group for CSF NfL and CSF p-tau181.

## Slide 3
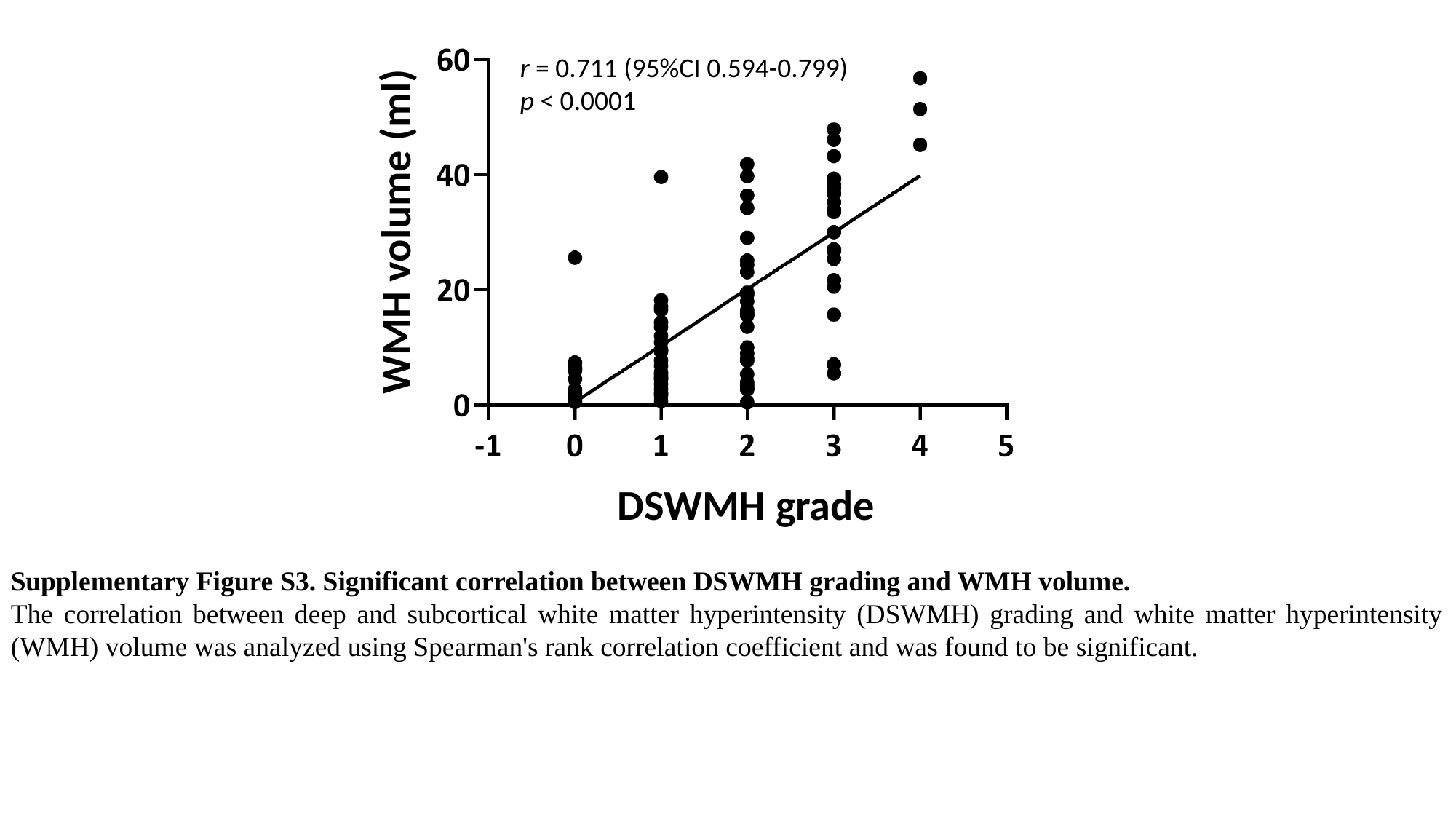

r = 0.711 (95%CI 0.594-0.799)
p < 0.0001
WMH volume (ml)
DSWMH grade
Supplementary Figure S3. Significant correlation between DSWMH grading and WMH volume.
The correlation between deep and subcortical white matter hyperintensity (DSWMH) grading and white matter hyperintensity (WMH) volume was analyzed using Spearman's rank correlation coefficient and was found to be significant.

## Slide 4
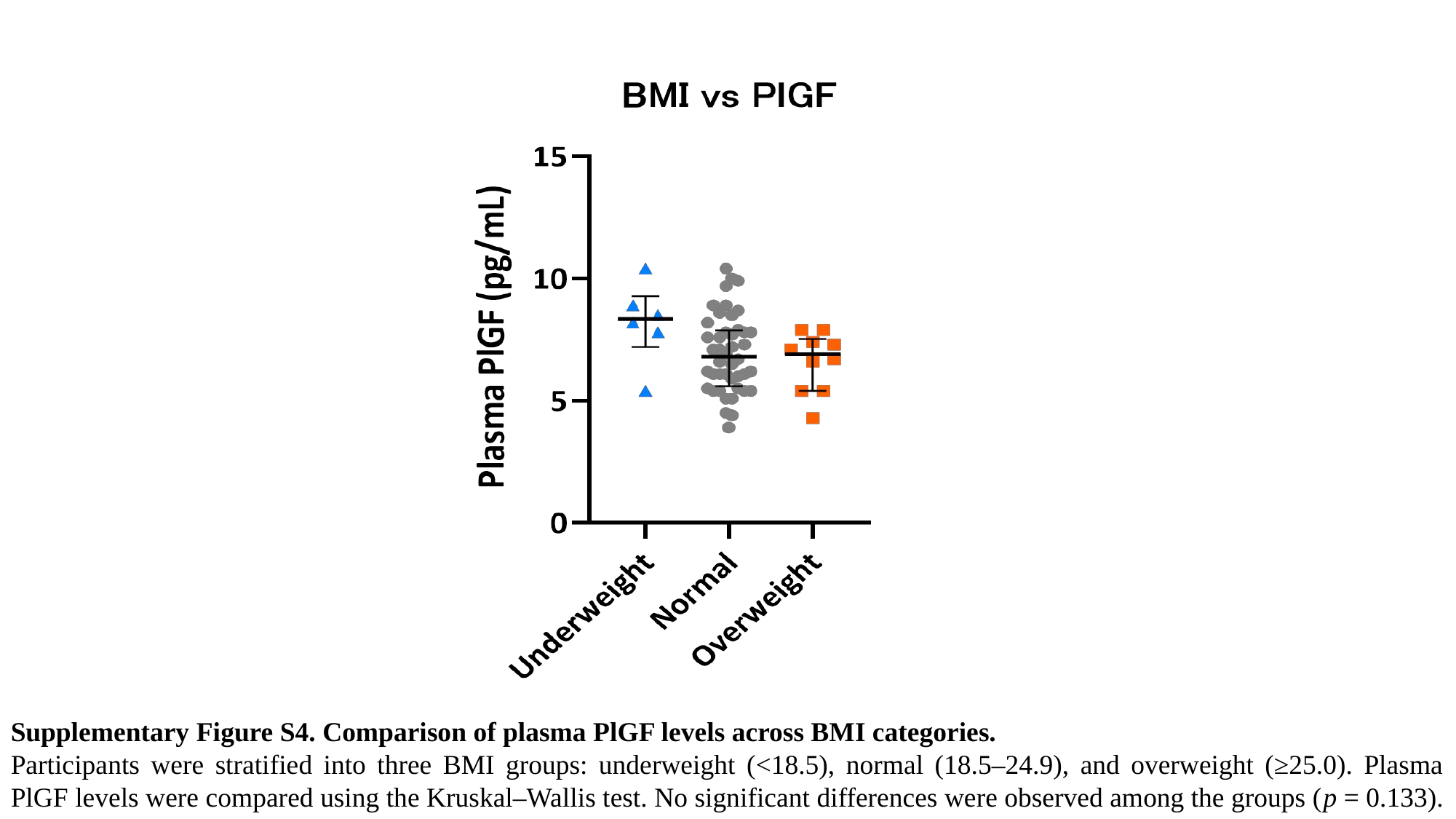

Supplementary Figure S4. Comparison of plasma PlGF levels across BMI categories.
Participants were stratified into three BMI groups: underweight (<18.5), normal (18.5–24.9), and overweight (≥25.0). Plasma PlGF levels were compared using the Kruskal–Wallis test. No significant differences were observed among the groups (p = 0.133).
