# Supplementary material for: Association of Plasma Placental Growth Factor with White Matter Hyperintensities in Alzheimer’s Disease
Source: Biomolecules. 2025 Sep 26;15(10):1367. doi: 10.3390/biom15101367 (PMC12564238; doi:10.3390/biom15101367)
Supplement: Supplementary file 1 [file biomolecules-15-01367-s001.zip › PlGF_Supplementary_Table_20250829.pdf]

Supplementary Table S1. MRI imaging parameters at each facility

Facility	A	B	C	D
**Vendor**	PHILIPS	SIEMENS	SIEMENS	Canon
**Model**	Ingenia	MAGNETOM Spectra	MAGNETOM Avanto Fit	Vantage Elan
**Magnetic field strength (T)**	1.5	3	1.5	1.5
**2D FLAIR**				
**TR/TE/inversion time (ms)**	10000/120/2650	10000/94/2639	12000/116/2757	8000/140/2500
**Slice thickness (mm)**	5	5	6	2
**Gap (mm)**	1	gapless	gapless	0.4
**Flip angle (°)**	90	150	160	90
**Matrix**	320 × 215	384 × 210	320 × 224	280 × 160
**Field of view (cm)**	23	23	22	20
**Voxel size (mm)**	0.45 × 0.45 × 5	0.3 × 0.3 × 5	0.3 × 0.3 × 6	0.69 × 0.63 × 2
**3D T1**				
**TR/TE/inversion time (ms)**	2000/4.6/1100	1800/2.99/800	1700/3.79/800	17/5.5/0
**Slice thickness (mm)**	1.6	1	1.3	1.5
**Gap (mm)**	gapless	gapless	gapless	gapless
**Flip angle (°)**	10	10	15	20
**Matrix**	256 × 256	256 × 256	256 × 256	256 × 256
**Field of view (cm)**	23	24	23	23
**Voxel size (mm)**	0.9 × 0.9 × 0.8	0.9 × 0.9 × 1	0.4 × 0.4 × 1.3	0.89 × 0.89 × 1.5
